# Protective Effects of Non-Anticoagulant Activated Protein C Variant (D36A/L38D/A39V) in a Murine Model of Ischaemic Stroke

**DOI:** 10.1371/journal.pone.0122410

**Published:** 2015-04-01

**Authors:** Anna P. Andreou, Maria Efthymiou, Yao Yu, Helena R. Watts, Faruq H. Noormohamed, Daqing Ma, David A. Lane, James TB Crawley

**Affiliations:** 1 Centre for Haematology, Imperial College London, London, United Kingdom; 2 Section of Anaesthetics, Pain Medicine & Intensive Care, Imperial College London, London, United Kingdom

## Abstract

Ischaemic stroke is caused by occlusive thrombi in the cerebral vasculature. Although tissue-plasminogen activator (tPA) can be administered as thrombolytic therapy, it has major limitations, which include disruption of the blood-brain barrier and an increased risk of bleeding. Treatments that prevent or limit such deleterious effects could be of major clinical importance. Activated protein C (APC) is a natural anticoagulant that regulates thrombin generation, but also confers endothelial cytoprotective effects and improved endothelial barrier function mediated through its cell signalling properties. In murine models of stroke, although APC can limit the deleterious effects of tPA due to its cell signalling function, its anticoagulant actions can further elevate the risk of bleeding. Thus, APC variants such as APC(5A), APC(Ca-ins) and APC(36-39) with reduced anticoagulant, but normal signalling function may have therapeutic benefit. Human and murine protein C (5A), (Ca-ins) and (36-39) variants were expressed and characterised. All protein C variants were secreted normally, but 5-20% of the protein C (Ca-ins) variants were secreted as disulphide-linked dimers. Thrombin generation assays suggested reductions in anticoagulant function of 50- to 57-fold for APC(36-39), 22- to 27-fold for APC(Ca-ins) and 14- to 17-fold for APC(5A). Interestingly, whereas human wt APC, APC(36-39) and APC(Ca-ins) were inhibited similarly by protein C inhibitor (t_½_ - 33 to 39 mins), APC(5A) was inactivated ~9-fold faster (t_½_ - 4 mins). Using the murine middle cerebral artery occlusion ischaemia/repurfusion injury model, in combination with tPA, APC(36-39), which cannot be enhanced by its cofactor protein S, significantly improved neurological scores, reduced cerebral infarct area by ~50% and reduced oedema ratio. APC(36-39) also significantly reduced bleeding in the brain induced by administration of tPA, whereas wt APC did not. If our data can be extrapolated to clinical settings, then APC(36-39) could represent a feasible adjunctive therapy for ischaemic stroke.

## Introduction

Ischaemic stroke is a leading cause of mortality and morbidity worldwide. Tissue-plasminogen activator (tPA) can be administered as thrombolytic therapy to promote clot dissolution and reduce further ischemia if administered within 4.5 hours of stroke onset.[1] However, tPA has limitations, including increased risk of intracerebral bleeding, as well as the effects associated with reperfusion injury.[2] In murine models of stroke, tPA treatment can activate matrix metalloproteinases[3], disrupt the blood brain barrier and cause neuronal toxicity, which can counter its therapeutic benefits.[4] Treatments that limit such deleterious effects could be of major clinical importance.

**Fig 1:**
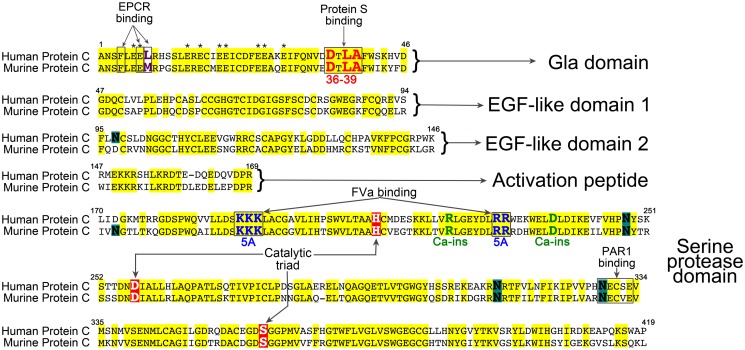
Amino acid alignment of human and murine protein C. Amino acid sequence is separated into different domains and numbered according to the human sequence. Amino acid identity is highlighted in yellow. Gla residues are denoted by *. Glycan attachment sites are highlighted in green. Residues involved in the catalytic triad are highlighted in red. Regions known to be important for protein S, factor Va and PAR1 binding are boxed. The amino acids mutated in each of the protein C variants 36–39, 5A and Ca-ins are shown in red, blue and green respectively.

The activated protein C (APC) anticoagulant pathway regulates thrombin generation through the proteolytic inactivation of cofactors FVa and FVIIIa.[5] The ability of APC to cleave FVa and FVIIIa is dependent upon distinct functional attributes. Inactivation of FVa and FVIIIa occurs on negatively-charged phospholipid surfaces. For this reason, APC anticoagulant function is dependent upon the phospholipid-binding Gla domain of APC.[6] The recognition of FVa by the serine protease domain of APC is partly dependent upon a positively charged loop adjacent to the active-site (Fig 1).[7] Similarly, a Ca^2+^-binding site in proximity to this loop in the serine protease domain is of great importance in the anticoagulant function of APC, likely by maintaining the conformation of the exosite(s) that recognise FVa.[8] Finally, in plasma the anticoagulant function of APC is almost entirely dependent upon its cofactor, protein S.[9] Protein S functions as a cofactor by increasing the affinity of APC for phospholipid surfaces and for FVa, and also through repositioning of the active site of APC that augments FVa and FVIIIa inactivation. The influence of protein S is primarily observed in the ~20 fold enhancement of the cleavage of FVa at R306. APC also exhibits cytoprotective cell signalling properties, primarily mediated through non-canonical proteolytic activation of protease activated receptor-1 (PAR1).[10,11] APC-dependent signalling to the endothelium elicits anti-inflammatory effects involving reduction in adhesion molecule expression, improvement of endothelial barrier function and anti-apoptotic effects. Through these actions, APC can limit the deleterious effects of tPA in murine models of stroke.[12,13] These beneficial effects are attributable to its cytoprotective function, rather than its anticoagulant activity. Indeed, the anticoagulant actions of APC appear to be detrimental in this model.[14,15] However, the potential increased likelihood of bleeding complications associated with co-administration of both a fibrinolytic and anticoagulant agent means there is necessary caution over the use of APC in stroke patients.[16] Thus, APC variants with reduced anticoagulant, but normal cytoprotective function may represent attractive adjunctive therapy options. Three such variants have been engineered (Fig 1); 1) APC KKK191-193AAA/RR229-230AA—termed APC(5A)—with serine protease domain mutations that impair FVa proteolysis, but do not influence PAR1 signalling [17], 2) APC R223C-D237C —termed APC(Ca-ins)—containing an engineered disulphide bond in a serine protease domain Ca^2+^-binding site that diminishes cleavage of FVa but not PAR1 [8], and 3) APC D36A/L38D/A39V —termed APC(36–39)—containing substitutions that abolish the essential protein S enhancement of APC anticoagulant function, but leaves its signalling function untouched [9,18].

A further variant APC KKK191-193AAA—termed APC(3A)—contains three of the five substitutions present in the APC(5A) variant. The impairment of anticoagulant function of this variant is not as severe as for APC(5A). However, APC(3A) is currently undergoing trials for use in humans with a view to exploring its efficacy as an adjunctive therapy to tPA in the setting of ischaemic stroke.[19]

We present here, the direct comparison of the anticoagulant functions and inactivation of the three non-anticoagulant APC variants, and an investigation of the therapeutic benefits of APC(36–39) in a murine model of ischaemic stroke.

## Material and Methods

### Production and quantitation of human and murine APC variants

The pRC/CMV/human protein C mammalian expression vector was used as template for site-directed mutagenesis to introduce the KKK191-193AAA/RR229-230AA (termed 5A), R223C/D237C (termed Ca-ins) and D36A/L38D/A39V (termed 36–39) mutations, as previously described (Fig 1).[20] The active site variant S360A was also generated. The mammalian expression vector pcDNA3.1 containing the murine protein C cDNA (gift from Prof John Griffin, The Scripps Research Institute, CA), was used as template for site-directed mutagenesis to introduce the corresponding mutations—KKK192-194AAA/RR230-231AA (5A), R224C/D238C (Ca-ins), E36A/L38D/A39V (36–39) and S360A —into murine protein C.[21] All mutations were verified by sequencing. HEK293 and HEK293T cells were used for stable and transient expression, respectively, of human and murine protein C variants, as previously described.[20] Vitamin K was added to culture medium to enable γ-carboxylation. For expression studies, HEK293T cells were transiently transfected with all vectors in parallel using PEI and thereafter, cultured in OptiMEM containing vitamin K for 3 days.[9] Thereafter, human and murine protein C expression and secretion was assessed by Western blot analysis of conditioned media and of cell lysates using polyclonal rabbit anti-human protein C (Dako) or sheep anti-mouse protein C (Haematologic Technologies Inc.), Similar analyses were also performed upon stably transfected HEK293 cells.

For purification of fully γ-carboxylated protein C human and murine protein C variants, 1-2L of conditioned media from each variant was concentrated ~20 fold by tangential flow filtration (Millipore), dialysed thoroughly into 20 mM Tris-HCl 150 mM NaCI, 10 mM EDTA, pH 7.4, and thereafter, purified using an anionic HiTrap Q Sepharose Fast Flow column (GE Healthcare). Samples were loaded in 20 mM Tris-HCl 150 mM NaCI, 10 mM EDTA, pH 7.4, washed with 20 mM Tris-HCl 150 mM NaCI, pH 7.4 and eluted with a step gradient into 20 mM Tris-HCl 150 mM NaCI, 50 mM CaCl_2_, pH 7.4, as previously published.[22] All fractions were analysed by Western blotting using polyclonal anti-human or anti-mouse protein C antibodies (1:1000; Haematologic Technologies Inc.) to assess isolation of protein C, and a mouse monoclonal anti-human Gla (1:1000; Haematologic Technologies Inc.) antibody to assess separation of γ-carboxylated and non-fully γ-carboxylated protein C.

Sandwich ELISAs for human and murine protein C were used to quantify protein C in all preparations. For this, 96-well Maxisorp plates (Nunc) were coated overnight with either sheep anti-human protein C (1:2000) or sheep anti-mouse protein C (1:1000; Haematologic Technologies Inc.) in 0.1M sodium carbonate (pH 9.6) buffer and blocked with 3% BSA in PBS for 2 hours. Human or murine protein C samples diluted in, 1% BSA in PBS, were added to wells. In parallel, a standard curve of 0–4 nM of human or murine plasma-derived protein C (Haematologic Technologies Inc.) was generated and incubated with wells. Thereafter, a rabbit anti-human-protein C-HRP antibody (1:2000; Dako) or a monoclonal rat anti-murine protein C antibody (1:1000; Haematologic Technologies Inc.) followed by a rabbit-anti-rat-HRP-antibody (1:3000; Dako) were used for detection.

Human protein C variants were activated with protac, as previously described[23], and repurified with a HiTrap Q-Sepharose FF column as before[9]. Murine protein C and its variants were activated using mouse thrombin and rabbit thrombomodulin (Haematologic Technologies Inc.) coupled to Dynabeads M-280 Tosylactivated (5 mg beads/100 μg thrombin-thrombomodulin for up to 9 μg/ml of protein C) for 5h at 37°C, according to the manufacturer’s instructions (Life Technologies). In both cases, confirmation of complete activation under these conditions was previously determined using an ELISA that is specific for protein C (and not APC) using monoclonal antibodies specific for either human or mouse protein C (Haematologic Technologies Inc.) as the capture antibody, and also based on the initial rate of hydrolysis of the S-2366 chromogenic substrate (Chromogenix).[24] For this, human or murine APC (and variants) were diluted in 20 mM Tris-HCl (pH 7.8), 150 mM NaCl, 2.5 mM CaCl_2_, 0.1mg/ml BSA and 0.1% polyethylene glycol (PEG 8000). To this, 400 μM (final concentration) of S-2366 substrate were added and hydrolysis of the S-2366 substrate measured by changes in absorbance at 405 nm. A standard curve was generated using 0–12 nM plasma derived human APC (Haematologic Technologies Inc.). Initial rates of proteolysis were plotted against APC concentration to generate the standard curve.

The amidolytic activity/catalytic efficiency of all human and murine APC variants was also assessed using the chromogenic substrate S2366 (Chromogenix) to determine kinetic constants k_cat_, K_m_ and k_cat_/K_m_, enabling APC concentrations for each variant to be determined in activated preparations through its enzymatic activity. For this, 2 nM of each fully activated APC preparation was incubated with a range of concentrations of S-2366 (0–2000 μM) in 20 mM Tris-HCl (pH 7.8), 150 mM NaCl, 2.5 mM CaCl_2_, 0.1mg/ml BSA and 0.1% PEG. The initial rate of the S-2366 hydrolysis was measured at 405 nm. Curve fitting was performed, using the Michaelis-Menten equation in GraphPad Prism. K_m_, V_max_, and k_cat_ parameters were calculated from which the k_cat_/K_m_ was derived. All samples were tested in duplicate and experiments were repeated three to four times.

### Binding of murine protein C variants to murine EPCR

The affinity of all murine protein C variants for murine EPCR was evaluated using a plate binding assay [63, 232]. For this, an anti-myc monoclonal antibody (1.5 μg/ml) (Sigma) in 0.1 M sodium carbonate (pH 9.6) was immobilised onto a 96-well plate at 4°C overnight. The plate was washed with TBS containing 5mM CaCl_2_, and 0.6mM MgCl_2_, followed by blocking with TBS-3% BSA. Soluble murine EPCR (5 μg/ml) with a C-terminal myc-His tag (a kind gift of Prof J Hermida, University of Navarra, Spain) [25] was added to each well and incubated at room temperature for 1 hour. After two washes with TBS containing 5mM CaCl_2_, and 0.6mM MgCl_2_, 100 μl of each murine protein C variant (0 to 1200 nM) diluted in TBS-1% BSA containing 5mM CaCl_2_, and 0.6mM MgCl_2_ were added in duplicate and incubated for 2 hours at 37°C with shaking. Following incubation, the plate was washed three times with TBS containing 5mM CaCl_2_, and 0.6mM MgCl_2_. Protein C-EPCR complexes were detected with addition of sheep anti-murine protein C followed by goat-anti-sheep-HRP. The amount of bound murine protein C was detected by addition of OPD and stopped by the addition of 50 μl of 3M H_2_SO_4_. Absorbance values were fitted using a one-site equation in GraphPad Prism 5.0. Apparent equilibrium binding constants (K_D(app)_) were calculated. Experiments were repeated three times.

### Inhibition of human APC variants by purified protein C inhibitor (PCI)

The rate of inactivation of 20nM human APC variants by 200nM human recombinant PCI (Prof J. Huntington, University of Cambridge, UK) was assessed by removing aliquots at selected time points (0–20 mins) and measuring residual APC activity based on the initial rate of proteolysis of the chromogenic substrate, S2366 (Chromogenix), to monitor active APC concentration.[26–28]

### Thrombin generation assays

The anticoagulant activities of the human APC variants were determined based on their ability to inhibit tissue factor (TF)-induced thrombin generation, using calibrated automated thrombography assays, as previously described.[9,29,30] Both normal pooled human plasma and protein C-deficient plasma (Affinity Biologicals) were used. For the assays, 80 μl of plasma, were incubated with 65 μg/ml corn trypsin inhibitor (HTI), 50 μM phospholipid vesicles (DOPS:DOPC:DOPE, 20:60:20), 4 pM human TF (Innovin, Dade Behring), and 0–20 nM APC, in a final volume of 100 μl (all concentrations are final). Thrombin generation was initiated by automatic dispensation of 20 μl of 2.5 mM Z-Gly-Gly-Arg-AMC·HCl (Bachem), 60 mg/ml BSA 100 mM CaCl_2_ in 20 mM Tris-HCl pH 7.4 into each well. The reactions were performed at 37°C. Measurements were taken at 20 second intervals for 40 minutes at wavelengths 390 nm (excitation) and 460 nm (emission) with a Fluoroscan Ascent FL Plate Reader (Thermo Lab System) in combination with the thrombinoscope software (Synapse, BV). A thrombin calibration standard (Synapse) was used to correct inner filter and substrate consumption effects. Thrombin generation was quantified by deriving the endogenous thrombin potential (ETP)—i.e. the area under the curve. All samples were tested in duplicate and experiments were repeated at least three times.

The ability of the murine APC variants to inhibit TF-induced thrombin generation was assessed in murine plasma (Innovative Research). Briefly, 40 μl of murine plasma was incubated with 65 μg/ml corn trypsin inhibitor, 50 μM phospholipid vesicles (DOPS:DOPC:DOPE, 20:60:20), 4 pM human TF, and (0 to 20 nM) murine APC in a final assay volume of 120 μl (all concentrations are final). Samples were tested as for human plasmas in duplicate and experiments were performed three times.

For evaluation of the relative anticoagulant activity of the human APC variants, the ETP generated in the absence of APC was taken as 100%, and % ETP plotted as a function of APC concentration. These were fitted using a one phase exponential decay non-linear regression equation to derived IC_50_ values for each APC variant.

### Murine transient middle cerebral artery occlusion (MCAO) model

All mouse procedures were performed in strict accordance with the UK Animals (Scientific Procedures) Act 1986 under a Home Office approved project license. All animal work was further approved by the Imperial College Committee on the Ethics of Animal Experiments. All surgery was performed under isoflurane anaesthesia, and all efforts were made to minimise suffering. The MCA of adult, male C57BL/6 mice, anaesthetised with 1–2.5% isoflurane in 30% oxygen enriched air, was occluded for 60 minutes using an occlusive intraluminal silicon-coated monofilament. Successful occlusion and reperfusion were confirmed by recording cerebral blood flow using laser Doppler flowmetry (Moor Instruments). Core temperature was monitored via a rectal probe and maintained at 37 ±0.5°C using a homeothermic blanket (Harvard Apparatus). Arterial oxygenation, heart rate, and respiratory rate were monitored via a femoral probe pulse oximetry (STARR Life Sciences Corp) and in all experiments these parameters were within the range expected for isoflurane-anaesthetised mice. Three hours post-MCAO, mice were randomly assigned to i.v. administration of either a) 10 mg/kg recombinant human tPA (10% bolus, 90% 30 min infusion), b) 250 μg/kg murine APC (wt or 36–39), c) both tPA and murine APC (wt or 36–39), d) saline control. Following recovery, mice were held in a 37°C cage for 2 hours, and then to a room with a 12 hour light/dark cycle.

After 24 hours, focal neurological deficit was scored.[31] Infarct and hemisphere areas of sectioned brains stained with cresyl violet were measured. Corrected infarct area was calculated and expressed as a mean±SEM percentage of contralateral hemisphere.[32] Oedema was calculated by the ratio of the area of the infarcted hemisphere vs. contralateral hemisphere.[32] Haemoglobin levels in homogenized ischemic hemispheres were determined by a spectrophotometric assay using Drabkin’s reagent (Sigma).[33]

### Neurologic evaluation

Twenty-four hours after treatment, an observer blinded to group assignment evaluated the animals individually for both focal and general neurological deficits using two separate previously validated graduated scales, with higher scores indicating more severe injury. The focal neurological deficit scale comprised observing appearance of: hair (0–2), ears (0–2), eyes (0–4), posture (0–4), spontaneous activity (0–4), and epileptiform behaviour (0–12), body symmetry (0–4), gait (0–4), circling behaviour (0–4), climbing a 45° slope platform (0–4), forelimb symmetry (0–4), compulsory circling of forelimbs (0–4), and whisker response (0–4), as previously described.[31]

### Quantification of cerebral infarction and oedema ratio

Mice brains removed 24h post-treatment were cryoprotected before being cut in a cryostat at 30 μm and stained with cresyl violet (Sigma-Aldrich) and photographed (Olympus C2020). Digitised images of brain sections of the same plane were analysed by a blinded observer using ImageJ image software (NIH). For each animal, quantification of the infarcted area was performed on ten predetermined stereotaxic levels 300 μm apart encompassing the rostro-caudal extent of the lesion, ranging from Bregma +2.22 mm to—4.60 mm. The injury volume was calculated by multiplying the area of healthy tissue in mm^2^ for each hemisphere by the thickness of brain sections (30 μm) and distance between them, with the difference between the volumes for the healthy tissue of each hemisphere representing infarct area, expressed as mm^3^, as previously reported.[34] The oedema ratio was calculated indirectly and represented as a ratio of the area of the infarcted hemisphere vs. contralateral hemisphere, as previously reported.[33]

### Quantification of cerebral haemoglobin

To measure signs of intracerebral bleeding, haemoglobin levels in the ischaemic hemisphere of freshly extracted and homogenised brains were determined by a spectrophotometric assay using Drabkin’s reagent (Sigma-Aldrich).[35] A standard curve was obtained with known amounts of haemoglobin (Sigma-Aldrich).

### Statistical analysis

For the *in vivo* studies, Cronbach’s α was used to test the reliability of data within different treatment groups. Kruskall-Wallis one way analysis of variance followed by a Mann-Whitney test was used to evaluate the efficacy of treatments in the MCAO model. *P* values <0.05 were considered statistically significant.

## Results and Discussion

### Expression and activation of human and murine protein C variants

Three APC variants (human and murine) with reduced anticoagulant function, but normal, or near normal, cell signalling properties—APC(5A), APC(Ca-ins) and APC(36–39) were generated (Fig 1). All human and murine protein C variants were secreted well. However, consistent with a previous report [8], an estimated 5–20% of the human and murine protein C(Ca-ins) variant was secreted as disulphide-linked dimers as evidenced by detection of an additional ~120kDa band by Western blotting (which was not present for the other variants), and that disappeared upon reduction. These results suggest that a proportion of this protein C(Ca-ins) variant does not contain the intended engineered disulphide bond, which leaves surface exposed free cysteines available that can lead to dimerisation.

Large scale expression of all variants was performed followed by isolation of fully γ-carboxylated protein C by anion exchange chromatography. Efficient separation of γ-carboxylated and non-γ-carboxylated protein C was confirmed using an anti-Gla mAb, which revealed that the protein C that did not bind efficiently to the ion exchange column was recognised by anti-protein C antibodies, but was not immunoreactive with the anti-Gla antibody. Protein C eluted from the column with Ca^2+^ was immunoreactive with both anti-protein C and anti-Gla antibodies.

### Affinity of murine protein C variants for murine EPCR

**Fig 2:**
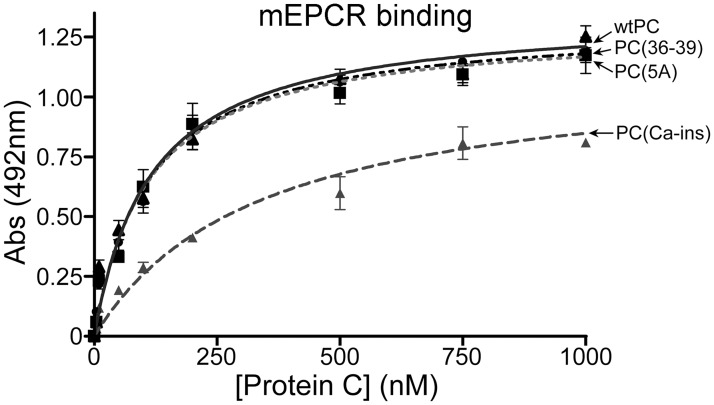
Binding of murine protein C variants to murine soluble EPCR. An anti-myc monoclonal antibody was immobilised onto a 96-well plate and used to capture soluble murine EPCR with a C-terminal myc-His tag. Murine protein C variants (0 to 1200 nM) diluted in TBS-1% BSA, 5mM CaCl_2_, 0.6mM MgCl_2_ were added in duplicate at 37°C with shaking. Protein C-EPCR complexes were detected with a sheep anti-murine protein C followed by goat-anti-sheep-HRP. Values (*n* = 3) represent the mean±SD.

Many of the important cytoprotective functions of APC are dependent upon its binding to EPCR prior to proteolytic activation of PAR-1.[36] Previous studies have revealed that the human APC(5A) and APC(36–39) variants have normal signalling function and/or EPCR binding.[17,18] Although the human APC(Ca-ins) variant harbours substitutions in the protease domain, which is spatially separated form the EPCR-binding site in the Gla domain, it has been reported that this confers reduced affinity for EPCR.[8] There are differences in the amino acid sequence of the ω-loop in the Gla domains of human and murine protein C that span the region that binds EPCR. This has revealed differences in the affinity of human protein C for murine EPCR, which may influence the potency of human APC in transducing cytoprotective signalling to the endothelium in mice.[37,38] This could warrant some caution when using human APC variants in murine models. To examine the effect of the non-anticoagulant substitutions upon the affinity of the murine protein C variants with murine EPCR, we performed a plate binding assay using the soluble extracellular domain of murine EPCR that was C-terminally tagged to enable immunocapture onto plates (Fig 2). Using this approach, we derived K_D(app)_ of 94.3nM ±18.3 nM of plasma-derived murine protein C for murine soluble EPCR. For the recombinant murine protein C variants, we derived K_D(app)_ of 112 ±17.6 nM for wt protein C, 110 ± 16.7 nM for protein C(5A), 111 ± 13.2 nM for protein C(36–39), 129 ± 17.1 nM for protein C(S360A), and 214 ± 30.4 nM for protein C(Ca-ins) (Fig 2). These results demonstrated that all murine variants exhibited normal affinity for murine soluble EPCR with the exception of the protein C(Ca-ins), which, similar to its human counterpart, had an approximately 2 fold reduced affinity for its receptor.

### Comparison of proteolytic function of human and murine APC variants

The activation of human protein C variants was performed using protac. All variants were activated normally, with the exception of protein C(Ca-ins), which was activated less efficiently (>2 fold less efficiently). This finding was similar to the findings of a previous report, which suggested that this protein C(Ca-ins) variant was also activated less well than wt protein C by the thrombin-thrombomodulin complex (although 60-80-fold better by thrombin alone).[8]

Activating the murine protein variants was not possible with protac, as this activator does not efficiently convert murine protein C. We attempted several different activation strategies including human thrombin, human thrombin in conjunction with rabbit soluble thrombomodulin, and murine thrombin with rabbit thrombomodulin. Of these, murine thrombin/rabbit thrombomodulin was the most efficient in converting the murine variants to APC, with the exception of the protein C(Ca-ins), which was more efficiently activated by murine thrombin alone in the presence of EDTA.

All human and murine protein C variants were fully activated prior to kinetic analysis of their proteolytic function using the APC chromogenic substrate S2366. Complete activation was confirmed using a protein C-specific ELISA capable of detecting any residual unactivated protein C in preparation. All human and murine APC variants exhibited very similar kinetic parameters (k_cat_ and K_m_) by comparison to wt APC. Of all the variants, only APC(Ca-ins) exhibited a slight (~25% increase) difference in K_m_ for proteolysis of the short substrate, S2366 (Table 1).

**Table 1:** Catalytic efficiencies (k_cat_/K_m_) of human (h) and murine (m) APC and variant APC-mediated proteolysis of the peptide substrate, S2366.

APC	kcat/Km (μM^-1^s^-1^)
hWT	0.22 ±0.02
hAPC(5A)	0.20 ±0.01
hAPC(36–39)	0.22 ±0.01
hAPC(Ca-ins)	0.17 ±0.02
mWT	0.22 ±0.03
mAPC(5A)	0.21 ±0.01
mAPC(36–39)	0.23 ±0.01
mAPC(Ca-ins)	0.17 ±0.02

### Direct comparison of APC variant anticoagulant function

**Fig 3:**
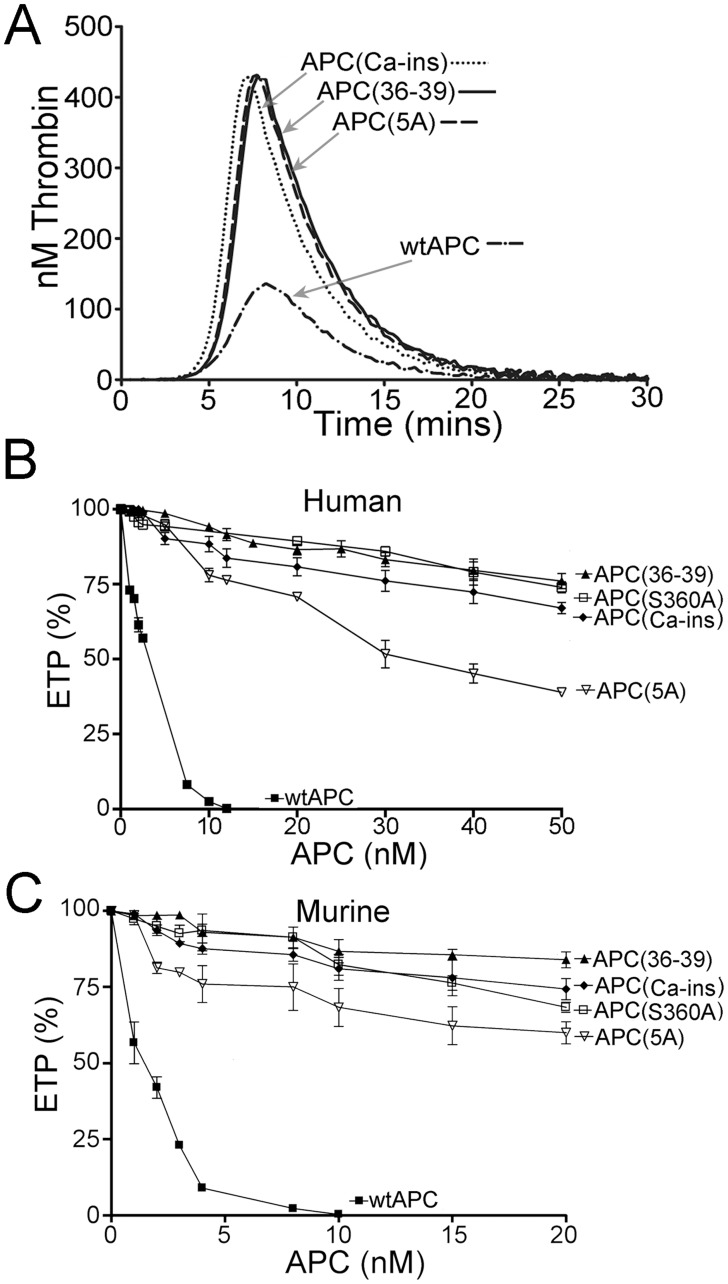
**A.** Representative example of thrombin generation in normal human plasma initiated with 4 pM TF measured in the presence of 2.5 nM wt APC or non-anticoagulant APC variants. **B-C.** Inhibition of TF-induced thrombin generation with increasing concentrations of either human (**B**) or murine (**C**) APC variants. The ETP in the absence of APC was taken as 100% and the ETP generated in the presence of each human (0 to 50 nM) or murine (0 to 20 nM) APC variant was expressed as a percentage of this. Values (*n* = 4) represent the mean±SD.

The anticoagulant actions of APC(36–39), APC(5A), or APC(Ca-ins) have previously been reported separately using quite different assays to monitor function.[8,9,17] For this reason, we sought to make a direct comparison of their relative anticoagulant activities using plasma-based thrombin generation assays. Using 2.5nM human APC(36–39), APC(5A), or APC(Ca-ins), very little or no effect on thrombin generation was observed compared to wt APC, which exhibited approximately 50% reduced ETP at this concentration (Fig 3A). This demonstrated that all variants had appreciably impaired anticoagulant function. Further titration of wt and variant human APC (up to 50nM) was performed and concentration dependent changes upon ETP were assessed. At 10nM wt human APC, almost complete inhibition of thrombin generation was achieved (Fig 3B). Even at 20nM however, all of the APC variants exhibited rather modest effects upon ETP. At 50nM, APC(5A) reduced ETP by approximately 50%, APC(Ca-ins) by ~30% and APC(36–39) by ~20%. Extrapolation of these results enabled estimation of IC_50_ values for each variant. For wt APC, this was 3.2 ±0.7nM, very similar to previous reports.[9,18] For APC(5A), APC(Ca-ins) and APC(36–39) these values were 43.8 ±5.6nM, 68.3 ±14.2nM and 158.0 ±41.5nM, respectively, consistent with their severely reduced anticoagulant activity. This corresponds to 14-, 22- and 50-fold reductions in anticoagulant function compared to wt APC, respectively.

We also performed similar analyses using the murine APC(5A), (Ca-ins) and (36–39) variants in murine plasma and titrating up to 20nM. These experiments revealed very similar results to those obtained using the equivalent human APC variants in human plasma and indicated reductions in anticoagulant function of 19-, 27 and 57-fold by comparison to murine wt APC (Fig 3C).

Taken together, these results demonstrate that all non-anticoagulant APC variants (human and murine) exhibit large reductions in their ability to function as anticoagulants, and suggest that, of these, the APC(36–39) has the most marked reduction in its ability to inhibit thrombin generation in plasma.

We originally aimed to generate and characterise the non-anticoagulant forms of APC with a view to exploring their therapeutic benefit in protecting against the deleterious effects of tPA in a murine model of stroke, and to examine whether they reduce the risk of bleeding associated with the use of wt APC. Similar studies have been carried out using APC(5A) and another variant, termed APC(3A), that contains just three (KKK191-193AAA) of the five substitutions present in the 5A form. Although murine APC(3A) exhibits a reduction in anticoagulant function (~10-fold *in vivo*), this is not as severe as for the murine APC(5A) variant in murine plasma. Both APC(5A) and APC(3A) have been shown to be efficacious in rodent models of stroke. Based on these studies APC(3A) is currently undergoing development and testing in humans.[12,14,19,37]

### Direct comparison of APC variant inactivation

**Fig 4:**
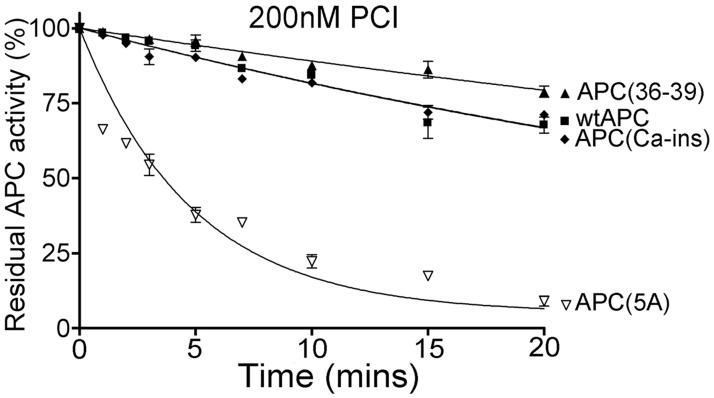
Time course of inhibition of human APC variants by PCI. APC variants (20 nM) were incubated with 200 nM PCI at 37°C. At designated time points (0–20 minutes), hydrolysis of 400 μM S-2366 substrate was used to determine of the concentration of residual APC. The graph represents the percentage of residual APC activity over time and values (*n* = 3) represent the mean±SD.

There exist some important species differences in the protein C system between humans and mice (Fig 1). For example, murine protein S does not appear to function efficiently as a cofactor for human APC.[39] Consequently, as APC function in plasma appears to be highly dependent upon protein S for its anticoagulant function, this suggests that the use of human APC (or variants thereof) in mouse models may naturally confer appreciably reduced anticoagulant activity when used in this setting. Studies using human APC in murine plasma have indicated that it exhibits significantly reduced anticoagulant function when compared to murine APC. A further potential difference between species may be in the inactivation of APC. In humans, APC has a plasma half-life of ~23 minutes.[40] PCI is just one of the inhibitors in human plasma capable of inhibiting APC. Intriguingly, this serpin is not present in murine plasma and, therefore, is not a determinant of its inactivation or functional half-life in mice. A previous report demonstrated that the positively charged loop that has been mutated in the APC(5A) and APC(3A) variants has a negative effect upon inhibition of APC by PCI. We therefore examined the inactivation rates of the human APC variants (20nM) by 200nM human recombinant PCI (Fig 4). Under these conditions, the inactivation of wt APC and variants APC(36–39) and APC(Ca-ins) was comparable (t_1/2_ 33 to 39 minutes), and very similar to previous reports for wt APC. However, the inactivation of APC(5A) was appreciably accelerated (t_1/2_ ~4 minutes)—consistent with a previous report that explored the influence of K191, K192 and K193 upon PCI-mediated inactivation.[26] The other major inhibitor of APC in plasma, α1-antitrypsin, is unlikely influenced by the mutations in the APC variants, based on the reported structural requirements for its inactivation of APC.[41,42] The enhanced inactivation rate of APC(5A) by PCI is due to the positively charged residues adjacent to the active site (KKK191-193) in wt APC normally repelling the bait loop of PCI, making inactivation inefficient. However, when these residues are substituted for non-charged alanine in the APC(5A) and APC(3A) variants, this repulsive effect is lost leading to enhanced inactivation. These findings suggest that, in humans, the APC(5A) and APC(3A) variants may have the potential to get inactivated by PCI more efficiently than wt APC, APC(36–39) and APC(Ca-ins). In mice however, due to the lack of PCI in plasma, the influence of enhanced PCI inactivation would have no effect. A recent report detailing findings from a Phase I study of APC(3A) in humans suggested a half-life (a combination of both inactivation and clearance) of 16 mins (range 13 to 18 mins), which may be moderately shorter than a previous estimate of 23 mins for wt APC.[19,40] These findings likely reflect a modest influence of PCI upon the normal inactivation/clearance of APC in humans.

### Evaluation of murine APC(36–39) in a mouse model of ischaemic stroke

The *in vitro* analyses of wt APC and the APC(5A), APC(36–39) and APC(Ca-ins) variants suggested that APC(Ca-ins) exhibited certain disadvantages as a non-anticoagulant form of APC. This was based on the findings that it is expressed partly in a dimeric form, and it also exhibited a modest reduction in affinity for EPCR suggesting that its cytoprotective function may be similarly compromised. APC(5A) exhibited normal expression, activation, EPCR binding and enzymatic function. However, based on the titration of this variant into thrombin generation assays, although it exhibited severely reduced anticoagulant function, APC(Ca-ins) and APC(36–39) appeared to be more profoundly compromised.

**Fig 5:**
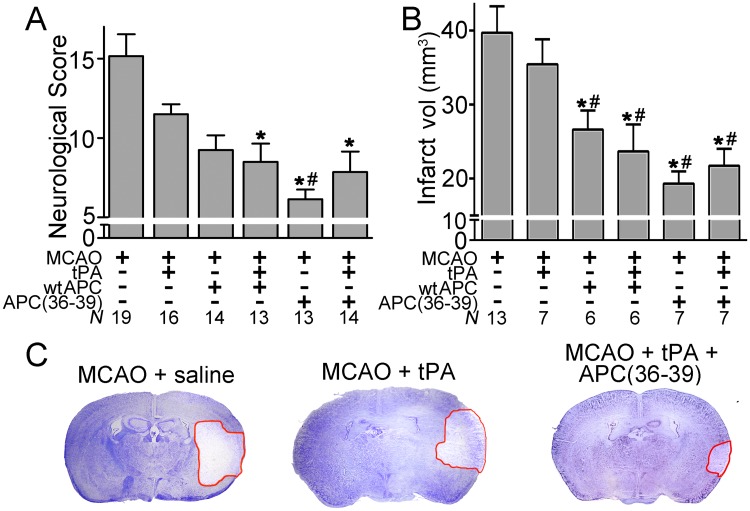
Effect of murine wt APC or APC(36–39) with or without tPA upon functional recovery and infarct lesion at 24h post-MCAO. Male mice were subject to transient MCAO for 60 min. tPA (10mg/kg), murine wt APC or APC(36–39) (250μg/kg) either alone or in combination with tPA, or saline control, were i.v. infused 3 hours post MCAO. **A.** Neurological deficit scoring of MCAO treated animals at 24 hours. **B.** Infarct volume (mm^3^) in brains from animals receiving different treatments at 24 hours **C.** Representative cresyl violet staining of brain coronal sections of ischemic mice treated with vehicle (saline), tPA or tPA + APC(36–39). Infarct area is denoted by the red line. All values are expressed as mean ± SEM. * *P* < 0.05 compared to mice treated with vehicle. # *P* < 0.05 compared to mice treated with tPA.

Therefore, based on our *in vitro* findings, we compared the therapeutic efficacy of wt APC and APC(36–39) both with and without tPA in the murine MCAO model. At 24-hours post treatment, mice were scored by a blinded observer using a defined focal neurological deficit scale. Following MCAO, mice receiving saline had a mean neurological score of 15.2 ±1.4 (n = 19). Mice that received 250μg/kg APC(36–39) alone, or in combination with 10mg/kg tPA, had significantly improved neurological scores (6.1 ±0.6; n = 13, and 7.9 ±1.3; n = 14, p <0.05, respectively), that was at least as effective as wt APC in improving this parameter (Fig 5A).

After 24 hours, mice were sacrificed and brain infarct volumes were determined in coronal sections (Fig 5B and 5C). After 24 hours post-MCAO, the infarct volume was 39.7mm^3^ ±3.6 (n = 13) in mice that received saline. The infarct area was similar (35.5mm^3^ ±3.4) in mice receiving tPA alone (n = 7). Significant reductions in infarct volume were observed in mice receiving wt APC both with (n = 6; p<0.05) and without tPA (n = 6; p<0.05). However, the largest reductions in infarct volume were for APC(36–39) alone and APC(36–39) in combination with tPA 19.3±1.7 mm^3^ (n = 7, p = 0.006) and 21.7±2.3 mm^3^ (n = 7, p = 0.03), respectively, representing an approximate 50% reduction in infarct volume by comparison to treatment with tPA alone.

**Fig 6:**
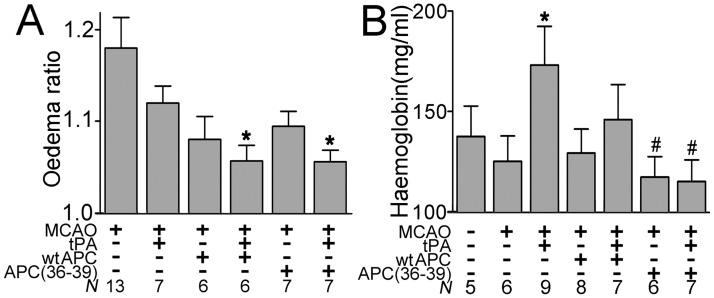
**A.** Influence of murine wt APC and APC(36–39) alone or in combination with tPA on the oedema ratio (area of the infarcted hemisphere vs. non-infarcted contralateral hemisphere). **B.** Haemoglobin levels in the ischaemic hemisphere of mice treated with murine wt APC or APC(36–39) ±tPA. All values are expressed as mean ± SEM. * *P* < 0.05 compared to mice treated with vehicle. # *P* < 0.05 compared to mice treated with tPA.

Using the ratio of the total area of the infarcted to non-infarcted brain hemisphere as a measure of brain oedema, APC(36–39) in combination with tPA significantly reduced brain oedema (n = 7; Fig 6A) to a similar extent as wt APC in combination with tPA (n = 6). These findings, with respect to the extent of oedema reduction, are similar to those reported in previous murine studies using the APC(3A) variant [15,37].

The effect of wt APC and APC(36–39) upon intracerebral haemorrhage was examined by measuring the concentrations of haemoglobin in homogenised infarcted brain hemispheres. Similar to previous reports using the MCAO model, tPA alone significantly increased signs of bleeding (haemoglobin 1.73±0.19mg/ml; n = 7, p<0.05) in the brain 24 hours post-treatment, by comparison to saline control (Fig 6B). Co-administration of tPA with APC(36–39) completely prevented the tPA-induced increase in bleeding (haemoglobin 1.15±0.10mg/ml; n = 7, p<0.05), which was not significantly different from haemoglobin levels in uninjured brains (haemoglobin 1.36±0.15mg/ml; n = 6). Although the mean haemoglobin concentration in homogenised infarcted brains from mice receiving tPA with APC(36–39) (1.15 ±0.10mg/ml; n = 7) was lower than in those mice receiving tPA with wt APC (1.46 ±0.17 mg/ml; n = 7), this did not reach statistical significance.

Taken together, our data suggest that the APC(36–39) variant may represent potential adjunctive therapy to tPA for the treatment of ischaemic stroke. They also demonstrate that targeting the protein S cofactor enhancement of APC is a viable strategy for producing non-anticoagulant APC. APC(3A) has currently undergone phase I testing in humans. The results from this small study revealed that APC(3A) was well tolerated in healthy individuals up to doses of 540μg/kg. At this dose, plasma concentrations of ~65nM APC were measured at 15 mins. Based on the reported functional half-life of 15 mins for APC(3A), the initial plasma concentrations of this dose might be predicted to be approximately 130nM. Although the APC(3A) variant has impaired anticoagulant function, this is reportedly about 10-fold reduced. Based on this figure, a concentration of 130nM APC(3A) may exert an anticoagulant action that approximates to that of 13nM wt APC. In plasma-based thrombin generation assays, 13nM APC is enough to completely abolish thrombin generation and could be considered to represent quite potent anticoagulation. In the phase I study, the degree of anticoagulation was measured at 1 hour post administration of APC(3A). Although significant dose-dependent increases in aPTT were reported, these increases at 1 hour were not considered likely to elevate the risk of bleeding. However, by 1 hour post-administration, APC(3A) has already been through four half-lives, which, by extrapolation, could suggest that the level of anticoagulation that may occur during the period immediately after infusion of the drug may be appreciably higher. It should be pointed out though, that whether this is likely to significantly alter bleeding risk, or bleeding severity is unclear at this time. However, this may provide a rationale to exploring the use of an APC variant with a more profound reduction in anticoagulant function than that of the APC(3A) variant.

At this time, it is difficult to ascertain the optimal dosing of human APC (and variants thereof) that are required for effective therapeutic cytoprotection. Based on our findings though, APC(36–39) may enable higher dosing than that of APC(3A) without appreciably perturbing the anticoagulant profile of the patient. Interestingly, a recent study by Ni Ainle *et al* suggested that alteration of N-linked glycosylation of the protein C serine protease domain through introduction of an Asn329Gln substitution significantly augmented APC cytoprotective signalling.[43] This finding may provide a further opportunity to create variants not only with reduced anticoagulant function, but also selectively enhanced cell signalling function. This later approach may, in turn, enable reduced levels of APC to be administered whilst maintaining therapeutic benefit. Although further studies are now required to explore the full potential of APC as an adjunct therapy in the setting of ischaemic stroke in human, our data, in combination with those from others, provide potential strategies to further augment the therapeutic efficacy of APC.

## References

[1] Lansberg MG, Schrooten M, Bluhmki E, Thijs VN, Saver JL (2009) Treatment time-specific number needed to treat estimates for tissue plasminogen activator therapy in acute stroke based on shifts over the entire range of the modified Rankin Scale. Stroke 40: 2079–2084. 10.1161/STROKEAHA.108.540708 19372447 PMC2881642

[2] Albers GW, Bates VE, Clark WM, Bell R, Verro P, Hamilton SA (2000) Intravenous tissue-type plasminogen activator for treatment of acute stroke: the Standard Treatment with Alteplase to Reverse Stroke (STARS) study. JAMA 283: 1145–1150. 10703776 10.1001/jama.283.9.1145

[3] Suzuki Y, Nagai N, Umemura K, Collen D, Lijnen HR Stromelysin-1 (MMP-3) is critical for intracranial bleeding after t-PA treatment of stroke in mice. J Thromb Haemost 5: 1732–1739. 17596135 10.1111/j.1538-7836.2007.02628.x

[4] Wang YF, Tsirka SE, Strickland S, Stieg PE, Soriano SG, Lipton SA (1998) Tissue plasminogen activator (tPA) increases neuronal damage after focal cerebral ischemia in wild-type and tPA-deficient mice. Nat Med 4: 228–231. 9461198 10.1038/nm0298-228

[5] Bouwens EA, Stavenuiter F, Mosnier LO (2013) Mechanisms of anticoagulant and cytoprotective actions of the protein C pathway. J Thromb Haemost 11 Suppl 1: 242–253. 10.1111/jth.12247 23809128 PMC3713536

[6] Zhang L, Jhingan A, Castellino FJ (1992) Role of individual gamma-carboxyglutamic acid residues of activated human protein C in defining its in vitro anticoagulant activity. Blood 80: 942–952. 1498334

[7] Gale AJ, Tsavaler A, Griffin JH (2002) Molecular characterization of an extended binding site for coagulation factor Va in the positive exosite of activated protein C. J Biol Chem 277: 28836–28840. 12063259 10.1074/jbc.M204363200

[8] Bae JS, Yang L, Manithody C, Rezaie AR (2007) Engineering a disulfide bond to stabilize the calcium-binding loop of activated protein C eliminates its anticoagulant but not its protective signaling properties. J Biol Chem 282: 9251–9259. 17255099 10.1074/jbc.M610547200

[9] Preston RJ, Ajzner E, Razzari C, Karageorgi S, Dua S, Dahlback B, et al (2006) Multifunctional specificity of the protein C/activated protein C Gla domain. J Biol Chem 281: 28850–28857. 16867987 10.1074/jbc.M604966200

[10] Mosnier LO, Zlokovic BV, Griffin JH (2007) The cytoprotective protein C pathway. Blood 109: 3161–3172. 17110453 10.1182/blood-2006-09-003004

[11] Mosnier LO, Sinha RK, Burnier L, Bouwens EA, Griffin JH (2012) Biased agonism of protease-activated receptor 1 by activated protein C caused by noncanonical cleavage at Arg46. Blood 120: 5237–5246. 10.1182/blood-2012-08-452169 23149848 PMC3537315

[12] Cheng T, Petraglia AL, Li Z, Thiyagarajan M, Zhong Z, Wu Z, et al (2006) Activated protein C inhibits tissue plasminogen activator-induced brain hemorrhage. Nat Med 12: 1278–1285. 17072311 10.1038/nm1498

[13] Liu D, Cheng T, Guo H, Fernandez JA, Griffin JH, Song X, et al (2004) Tissue plasminogen activator neurovascular toxicity is controlled by activated protein C. Nat Med 10: 1379–1383. 15516929 10.1038/nm1122

[14] Wang Y, Zhang Z, Chow N, Davis TP, Griffin JH, Chopp M, et al (2012) An activated protein C analog with reduced anticoagulant activity extends the therapeutic window of tissue plasminogen activator for ischemic stroke in rodents. Stroke 43: 2444–2449. 10.1161/STROKEAHA.112.658997 22811462 PMC3429704

[15] Guo H, Singh I, Wang Y, Deane R, Barrett T, Fernandez JA, et al (2009) Neuroprotective activities of activated protein C mutant with reduced anticoagulant activity. Eur J Neurosci 29: 1119–1130. 10.1111/j.1460-9568.2009.06664.x 19302148 PMC2692517

[16] Bernard GR, Vincent JL, Laterre PF, LaRosa SP, Dhainaut JF, Lopez-Rodriguez A, et al (2001) Efficacy and safety of recombinant human activated protein C for severe sepsis. N Engl J Med 344: 699–709. 11236773 10.1056/NEJM200103083441001

[17] Mosnier LO, Yang XV, Griffin JH (2007) Activated protein C mutant with minimal anticoagulant activity, normal cytoprotective activity, and preservation of thrombin activable fibrinolysis inhibitor-dependent cytoprotective functions. J Biol Chem 282: 33022–33033. 17872949 10.1074/jbc.M705824200

[18] Harmon S, Preston RJ, Ni Ainle F, Johnson JA, Cunningham MS, Smith OP, et al (2008) Dissociation of activated protein C functions by elimination of protein S cofactor enhancement. J Biol Chem 283: 30531–30539. 10.1074/jbc.M802338200 18779332 PMC2662146

[19] Lyden P, Levy H, Weymer S, Pryor K, Kramer W, Griffin JH, et al (2013) Phase 1 safety, tolerability and pharmacokinetics of 3K3A-APC in healthy adult volunteers. Curr Pharm Des 19: 7479–7485. 24372304 10.2174/1381612819666131230131454 PMC4040367

[20] Preston RJ, Villegas-Mendez A, Sun YH, Hermida J, Simioni P, Philippou H, et al (2005) Selective modulation of protein C affinity for EPCR and phospholipids by Gla domain mutation. Febs J 272: 97–108. 15634335 10.1111/j.1432-1033.2004.04401.x

[21] Mosnier LO, Gale AJ, Yegneswaran S, Griffin JH (2004) Activated protein C variants with normal cytoprotective but reduced anticoagulant activity. Blood 104: 1740–1744. 15178575 10.1182/blood-2004-01-0110

[22] Yan SC, Razzano P, Chao YB, Walls JD, Berg DT, McClure DB, et al (1990) Characterization and novel purification of recombinant human protein C from three mammalian cell lines. Biotechnology (N Y) 8: 655–661. 1366628 10.1038/nbt0790-655

[23] Zhang L, Castellino FJ (1990) A gamma-carboxyglutamic acid (gamma) variant (gamma 6D, gamma 7D) of human activated protein C displays greatly reduced activity as an anticoagulant. Biochemistry 29: 10828–10834. 2125495 10.1021/bi00500a016

[24] Yang L, Manithody C, Rezaie AR (2002) Contribution of basic residues of the 70-80-loop to heparin binding and anticoagulant function of activated protein C. Biochemistry 41: 6149–6157. 11994010 10.1021/bi015899r

[25] Lopez-Sagaseta J, Montes R, Hermida J (2009) Recombinant expression of biologically active murine soluble EPCR. Protein Expr Purif 64: 194–197. 10.1016/j.pep.2008.11.002 19041722

[26] Friedrich U, Blom AM, Dahlback B, Villoutreix BO (2001) Structural and energetic characteristics of the heparin-binding site in antithrombotic protein C. J Biol Chem 276: 24122–24128. 11316800 10.1074/jbc.M011567200

[27] Li W, Adams TE, Nangalia J, Esmon CT, Huntington JA (2008) Molecular basis of thrombin recognition by protein C inhibitor revealed by the 1.6-A structure of the heparin-bridged complex. Proc Natl Acad Sci U S A 105: 4661–4666. 10.1073/pnas.0711055105 18362344 PMC2290767

[28] Berg DT, Gerlitz B, Shang J, Smith T, Santa P, Richardson MA, et al (2003) Engineering the proteolytic specificity of activated protein C improves its pharmacological properties. Proc Natl Acad Sci U S A 100: 4423–4428. 12671072 10.1073/pnas.0736918100 PMC153571

[29] Ahnstrom J, Andersson HM, Hockey V, Meng Y, McKinnon TA, Hamuro T, et al (2012) Identification of functionally important residues in TFPI Kunitz domain 3 required for the enhancement of its activity by protein S. Blood 120: 5059–5062. 10.1182/blood-2012-05-432005 23074276

[30] Ahnstrom J, Andersson HM, Canis K, Norstrom E, Yu Y, Dahlback B, et al (2011) Activated protein C cofactor function of protein S: a novel role for a gamma-carboxyglutamic acid residue. Blood 117: 6685–6693. 10.1182/blood-2010-11-317099 21508412

[31] Homi HM, Yokoo N, Ma D, Warner DS, Franks NP, Maze M, et al (2003) The neuroprotective effect of xenon administration during transient middle cerebral artery occlusion in mice. Anesthesiology 99: 876–881. 14508320 10.1097/00000542-200310000-00020

[32] Swanson RA, Morton MT, Tsao-Wu G, Savalos RA, Davidson C, Sharp FR (1990) A semiautomated method for measuring brain infarct volume. J Cereb Blood Flow Metab 10: 290–293. 1689322 10.1038/jcbfm.1990.47

[33] Choudhri TF, Hoh BL, Solomon RA, Connolly ES Jr., Pinsky DJ (1997) Use of a spectrophotometric hemoglobin assay to objectively quantify intracerebral hemorrhage in mice. Stroke 28: 2296–2302. 9368579 10.1161/01.str.28.11.2296

[34] Thiyagarajan M, Fernandez JA, Lane SM, Griffin JH, Zlokovic BV (2008) Activated protein C promotes neovascularization and neurogenesis in postischemic brain via protease-activated receptor 1. J Neurosci 28: 12788–12797. 10.1523/JNEUROSCI.3485-08.2008 19036971 PMC2742231

[35] Wang J, Rogove AD, Tsirka AE, Tsirka SE (2003) Protective role of tuftsin fragment 1–3 in an animal model of intracerebral hemorrhage. Ann Neurol 54: 655–664. 14595655 10.1002/ana.10750

[36] Riewald M, Petrovan RJ, Donner A, Mueller BM, Ruf W (2002) Activation of endothelial cell protease activated receptor 1 by the protein C pathway. Science 296: 1880–1882. 12052963 10.1126/science.1071699

[37] Guo H, Wang Y, Singh I, Liu D, Fernandez JA, Griffin JH, et al (2009) Species-dependent neuroprotection by activated protein C mutants with reduced anticoagulant activity. J Neurochem 109: 116–124. 10.1111/j.1471-4159.2009.05921.x 19166505 PMC2692505

[38] Sen P, Clark CA, Gopalakrishnan R, Hedner U, Esmon CT, Pendurthi UR, et al (2012) Factor VIIa binding to endothelial cell protein C receptor: differences between mouse and human systems. Thromb Haemost 107: 951–961. 10.1160/TH11-09-0672 22370814 PMC3883592

[39] Fernandez JA, Heeb MJ, Xu X, Singh I, Zlokovic BV, Griffin JH (2009) Species-specific anticoagulant and mitogenic activities of murine protein S. Haematologica 94: 1721–1731. 10.3324/haematol.2009.009233 19815836 PMC2791928

[40] Okajima K, Koga S, Kaji M, Inoue M, Nakagaki T, Funatsu A, et al (1990) Effect of protein C and activated protein C on coagulation and fibrinolysis in normal human subjects. Thromb Haemost 63: 48–53. 2140205

[41] Shen L, Dahlback B, Villoutreix BO (2000) Tracking structural features leading to resistance of activated protein C to alpha 1-antitrypsin. Biochemistry 39: 2853–2860. 10715104 10.1021/bi992357p

[42] Glasscock LN, Gerlitz B, Cooper ST, Grinnell BW, Church FC (2003) Basic residues in the 37-loop of activated protein C modulate inhibition by protein C inhibitor but not by alpha(1)-antitrypsin. Biochim Biophys Acta 1649: 106–117. 12818196 10.1016/s1570-9639(03)00164-x

[43] Ni Ainle F, O'Donnell JS, Johnson JA, Brown L, Gleeson EM, Smith OP, et al (2011) Activated protein C N-linked glycans modulate cytoprotective signaling function on endothelial cells. J Biol Chem 286: 1323–1330. 10.1074/jbc.M110.159475 21044954 PMC3020740

